# A Music Therapist Shares Stories of Patients with Cancer

**DOI:** 10.3390/cancers8110104

**Published:** 2016-11-15

**Authors:** Kathy Jo Gutgsell

**Affiliations:** Music Therapy Department, University Hospitals Cleveland Medical Center, Cleveland, OH 44106, USA; kathy.gutgsell@uhhospitals.org

Since the publication of my research reporting that music therapy reduces pain in palliative care patients [1], many people have expressed curiosity about what it is I do with patients. I am a board-certified music therapist, one of many thousands who practice in the U.S. and around the world, and I depend upon the growing body of research in our field to guide me in my choice of interventions to meet my patients’ clinical needs. These may include pain management [1,2,3,4], dealing with grief and loss [4], improving family communication [5], accessing memories and prior coping skills [6], creating opportunities for self-expression [7], and creating a legacy [6]. My work at University Hospitals Seidman Cancer Center in Cleveland, Ohio and one of its affiliates, Southwest Hospice, enables me to provide music therapy to people with life-threatening illnesses. I want to tell you about my patients and in doing so, honor their lives. We as therapists create a place of trust wherein patients are free to share their stories as they prepare for the final journey into death. In the transcendent capacity of the therapeutic relationship, we listen, and then we design individualized music therapy interventions to meet our patients’ needs.

To illustrate the intimacy that can quickly arise in the therapeutic relationship, let me tell you about Donald (all names have been changed to protect the privacy of the individuals), a 70 year old music-lover with metastatic colon cancer. I told him I was a music therapist, and as I brought my chair next to his bed, I invited him to share his story. Minutes later, before I had played a note of music, he shared his grief and loss with tears saying, “One of these days will be the last time I hear the Mozart Requiem”. He shared his suffering about what it meant to lose everyone and everything he loved. I provided compassionate presence and listened as he spoke, humbled and moved by the trust he placed in me. Knowing that he lived with his son, I asked if he had shared these feelings with him. He said that because he wanted to spare his son the hurt, he had not. I urged him to give his son the gift of intimacy that he had given me, but since Donald was discharged later that day, I will never know what transpired between them. I have had to learn to be comfortable with not knowing every outcome, for at times my role is to sow a seed, and not to witness the harvest.

In contrast, I will share the story of Mary.

I will never forget Mary, who died of cancer and left a husband and four adult children. Her relationship with her family had been fraught with tension. The word abuse was used, although I never learned the details. Mary told me that she loved butterflies and made wooden houses for them which she donated to the local city park. She knew the migration and wintering locations and was troubled by the decline of their numbers. Sensing an opening into a place of healing and improving family communication, and knowing the value and evidence base of a therapeutic song-writing intervention, I asked if she would like to write a song about butterflies. She eagerly agreed, and we composed her song over many sessions in her home. She was fascinated by the transformation of the caterpillar into a butterfly. For weeks, she tiptoed around the issues of change and forgiveness, but one day she said, “I wonder if I could ever transform?” We inserted her query into the lyric as well as all of her fascination about butterflies. I set her lyrics to music, and then sang it for her. With her approval, I recorded it and made copies for each member of her family along with copies of the lyrics. Mary died soon after, and I received a call from her priest asking if I would sing the song at her funeral. The family, deeply touched by Mary’s song, had given the recording to her priest. His eulogy was an in-depth exploration of Mary’s song. He believed the song was her attempt to heal her relationships with her family. It was not a neat and tidy ending to family reconciliation, but it meant the world to Mary’s family. It was all they had, and it was enough.

In end of life care, music therapists often help patients access important memories and prior coping skills. I remember an elderly woman from Ukraine named Aneta who spoke limited English, who, when asked to recall songs from her country, began to sing a lullaby in her native tongue that her mother sang to her as a child. As I began to sing with her, her eyes grew wide in surprise. “You know this?” I witnessed the peace that came over her face, a dying woman, far from her home country with no living relatives, able to recall the comfort of her mother singing to her. Research tells us that music connects us to memories, to solace, to home [2].

Jarod’s story is very different. By contrast, he was only 27 when he died of sarcoma. I asked Jarod to tell me about a time when he felt truly alive and connected to what mattered most to him. He told me about being in the woods playing drums with a group of his closest friends. He told me how close he felt to nature, his friends, and to his deepest self in that drum circle. When asked about doing it again, he looked around the sterile hospital room and rolled his eyes. I assured him that music lives everywhere, even in hospital rooms. In order to create an opportunity for Jarod to express himself, we were able to facilitate a drum circle, a commonly used music therapy intervention, with some of his friends, family, and his favorite nurses and doctors in his hospital room the week before he died. Even though he was weak and frail by that time, he revealed more vitality in that drum circle than we had seen in days. With Jarod’s permission, we recorded the event as a legacy for his girlfriend and daughters. A few days later, Jarod was scheduled to transfer to a hospice facility close to his home. I received an urgent call from the nurse manager that Jarod wanted to speak with me. I quickly made my way to his hospital unit, and there, waiting by the elevator, were Jarod and his girlfriend and the ambulance team. “We couldn’t leave without saying goodbye”, Jarod said as he extended his hand. “Thank you for what you did for me. I am going to be OK, one day at a time”.

Finally, I remember so fondly a 58 year-old woman named Donna. She had metastatic breast cancer, and the first time I met her, I was struck by her poise, dignity and fortitude in facing death. She possessed a strong Christian faith, and did not worry for herself. She crumpled, though, when she spoke of her daughter Crystal. She didn’t want to cause Crystal any pain by leaving her, for they were as close as best friends. When I suggested we write a song that would leave Crystal a legacy of her mother’s love, Donna’s face brightened. Donna shared stories, memories, impressions, and I took notes. Later, in my office, I arranged her words into verse, and inserted them into the melody of Donna’s favorite song. The next day when I sang it to her, she made some important corrections to the lyrics, and then agreed to sing it herself, even though she did not consider herself a singer. I recorded her right there in her hospital room, and gave her the CD and digital file that same day. Crystal was thrilled with her song. Donna later told me that writing the song-writing intervention helped her come to a peace of mind about dying. “Writing and singing that song helped me more than anything else”, for in sharing her song, she expressed what mattered to her the most.

All of our patients who live and die with cancer have unique stories to share. They come to our hospital with a lifetime of experiences and relationships and coping skills. I treasure the times I have been able to help my patients manage pain, cope with grief and loss, improve family communication, find peace of mind through accessing comforting memories, express themselves, and leave a legacy to loved ones. Through the power of the therapeutic relationship and selected evidenced-based music therapy interventions, I have been privileged to bear witness as my patients share their stories.
